# Supplementary material for: NAT10-mediated acetylation of NIK mRNA in B cells promotes IgA production
Source: EMBO Rep. 2025 Jul 4;26(15):3917–36. doi: 10.1038/s44319-025-00509-2 (PMC12331902; doi:10.1038/s44319-025-00509-2)
Supplement: Supplementary file 10 — Expanded View Figures [file 44319_2025_509_MOESM10_ESM.pdf]

Expanded View Figures

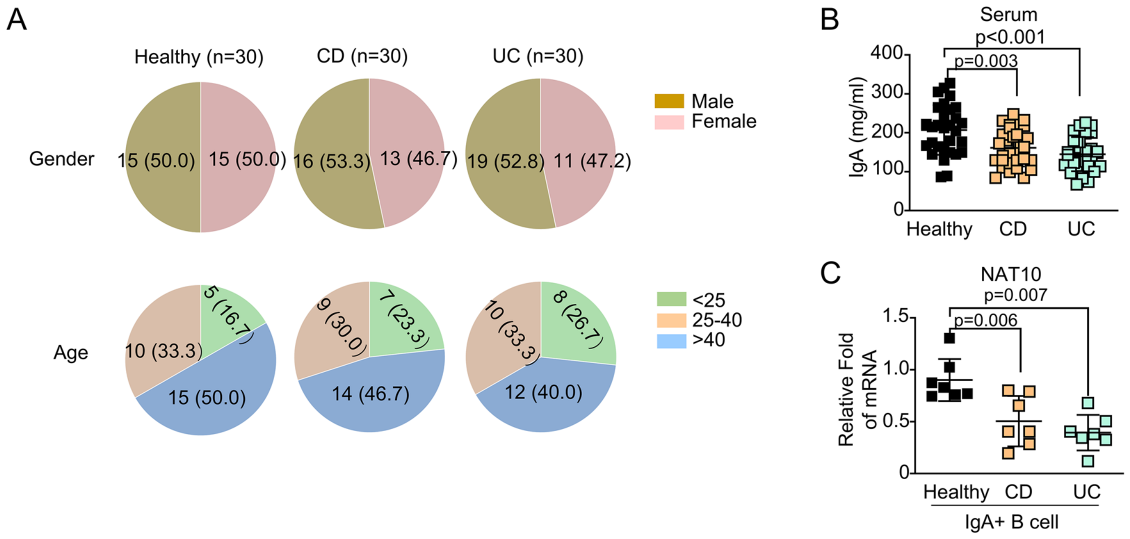

Figure EV1. Clinical information and grouping of IBD patients.

(**A**) Sex and age of patients with IBD and healthy volunteers ($n = 30$). (**B**) ELISA was used to quantify sIgA levels in serum from healthy donors and newly diagnosed patients with CD or UC ($n = 30$). (**C**) NAT10 expression was analyzed by qPCR in IgA$^+$CD19$^+$ B cells isolated from the colonic tissues of healthy donors and newly diagnosed CD or UC patients ($n = 7$). Results are shown as fold changes relative to *Actb* mRNA levels, normalized with Bio-Rad CFX Manager 3.1. All data are representative of biological replicates at least three independent experiments. Data are represented as the means ± SDs. The significance of differences (**B**, **C**) was determined using one-way ANOVA with Newman-Keuls post-hoc test. $^{**}P < 0.01$; $^{***}P < 0.005$. Source data are available online for this figure.

 © The Author(s)

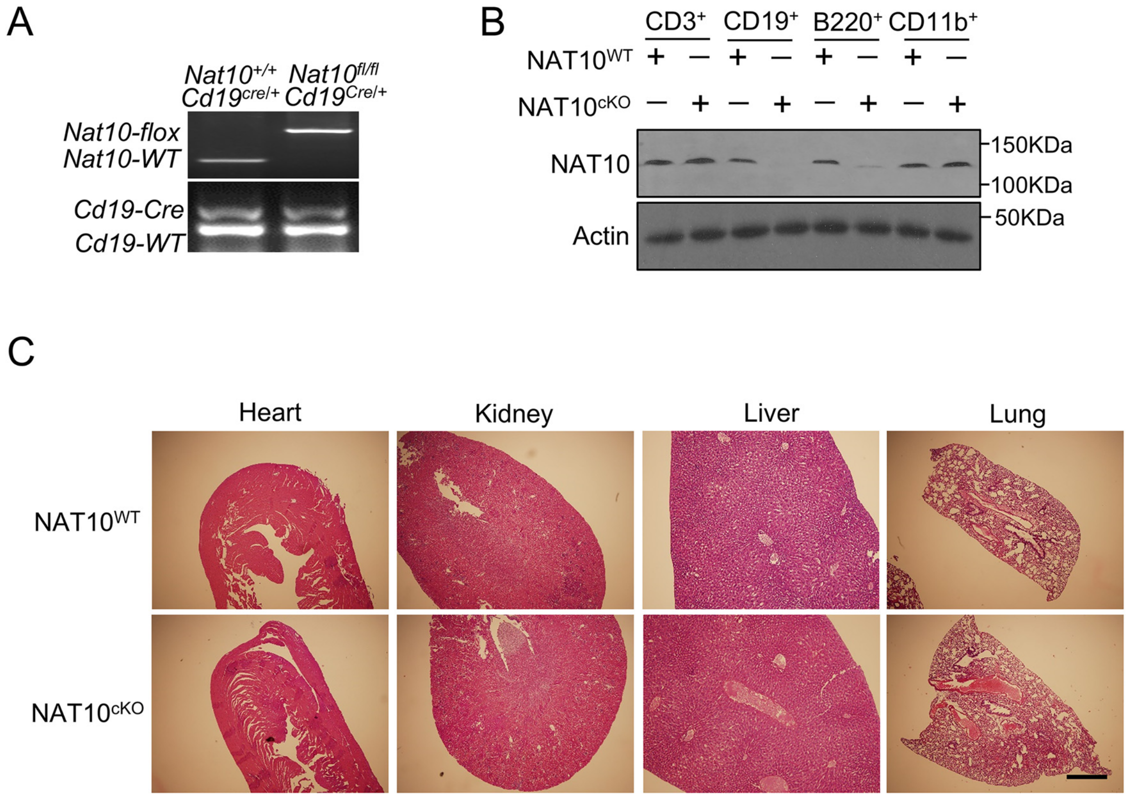

Figure EV2. Validation of NAT10 knockout efficiency in B cells.

(**A**) $NAT10^{cKO}$ genotyping PCR. Nat10-floxed mice were crossed with CD19-Cre mice to generate $Nat10^{fl/fl}Cd19^{Cre/+}$ ($NAT10^{cKO}$), and $Nat10^{+/+}Cd19^{Cre/+}$ (WT). (**B**) Immunoblotting (IB) assays showing specific ablation of NAT10 in the B cells of $NAT10^{cKO}$ mice. (**C**) Tissue sections of heart, kidney, liver, and lung from 6-week-old WT and $NAT10^{cKO}$ mice were performed with hematoxylin and eosin (H&E) staining ($n = 3$). All data are representative of biological replicates at least three independent experiments. Source data are available online for this figure.

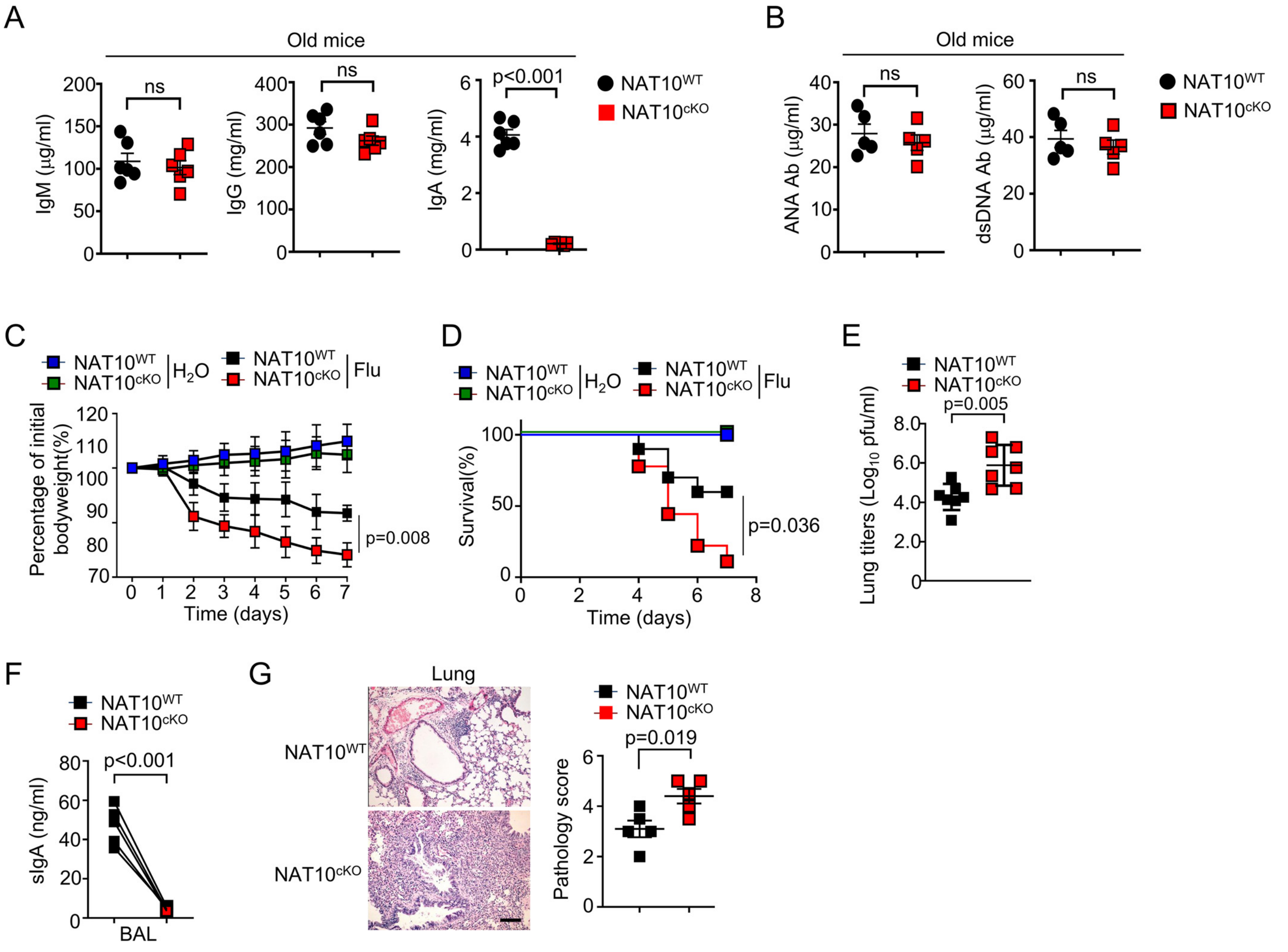

Figure EV3. Assessment of IgA levels in aged mice.

(**A**) Measurement of total antibody subclasses in the serum of 12-month-old WT and NAT10^{cKO} mice using ELISA ($n = 6$). (**B**) ELISA analysis of baseline levels of autoantibodies against antinuclear antigen (ANA Ab) and double-stranded DNA (dsDNA Ab) in the serum of 12-month-old unimmunized WT and NAT10^{cKO} mice ($n = 5$). (**C**) Body weight was tracked over a 7-day period in WT and NAT10^{cKO} mice that were intranasally administered either influenza virus H7N9 or PBS ($n = 7$ per group). (**D**) Survival rates of H7N9-infected WT and NAT10^{cKO} mice were monitored for 7 days ($n = 11$ per group). (**E**) Viral titers in the lungs were measured at 5 days post-infection (dpi) using a TCID50 assay ($n = 7$ per group). (**F**) ELISA was performed to assess IgA concentrations in the bronchoalveolar lavage fluid (BAL) at 5 dpi ($n = 5$). (**G**) Lung tissue sections from 6-week-old WT and NAT10^{cKO} mice infected with H7N9 were stained with H&E for histological analysis ($n = 5$). All data are representative of biological replicates at least three independent experiments. Data are represented as the means ± SDs. The significance of differences (**A**, **B**) and (**E**, **F**) was determined by *t* test, and those (**C**, **D**) were determined using one-way ANOVA with Newman–Keuls post-hoc test. $^{**}P < 0.01$; $^{***}P < 0.005$. Source data are available online for this figure.

 © The Author(s)

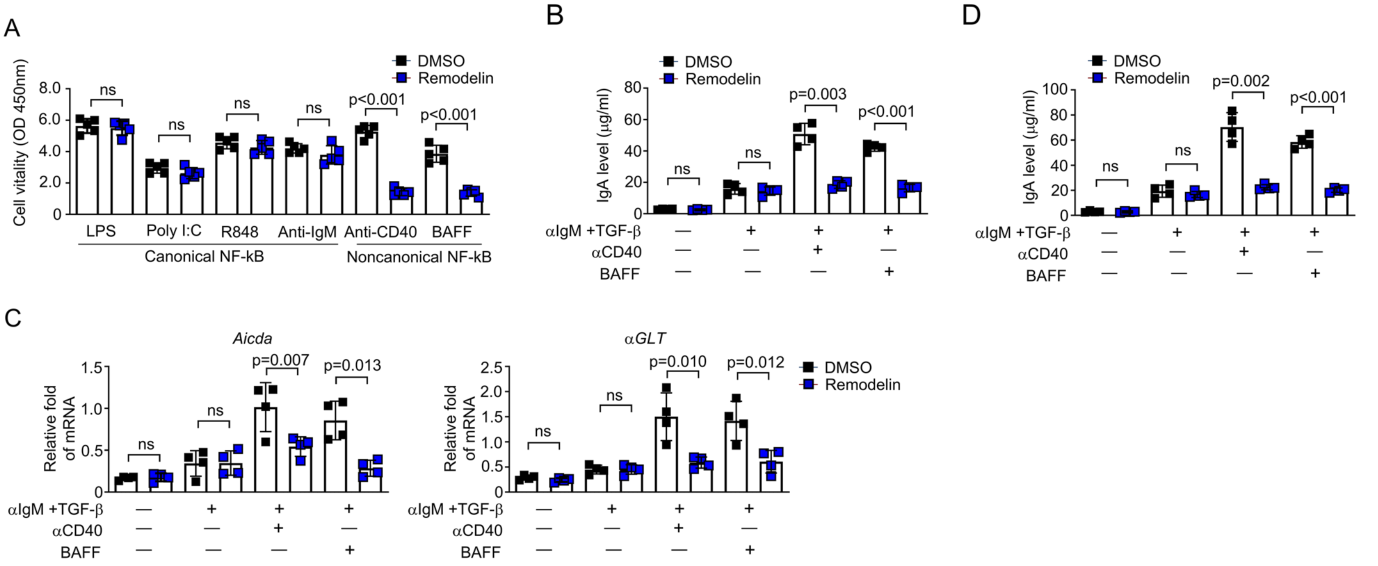

Figure EV4. NAT10 inhibitor, Remodelin, enhances IgA production via noncanonical NF-κB pathway.

(**A**) Proliferation assays of splenic B cells were incubated with complete medium containing 20 μM Remodelin, and stimulated with the specified inducers. These assays measured the proliferation of splenic B cells in response to the indicated stimuli ($n = 5$). (**B**) Intracellular IgA levels in splenic B cells were incubated with complete medium containing 20 μM Remodelin, and were analyzed after 5 days of culture with αIgM (10 μg/ml) alone or combined with TGF-β (2 ng/ml), anti-CD40 (α-CD40; 1 μg/ml), or BAFF (200 ng/ml). The percentage of IgA^+ B cells was quantified by flow cytometry, with numbers shown in the outlined regions ($n = 4$). (**C**) qPCR was performed to assess α-GLT and AID mRNA expression in splenic B cells after 5 days of culture under the same conditions as in (**B**). The results are presented as fold changes relative to *Actb* mRNA levels and normalized using Bio-Rad CFX Manager 3.1 ($n = 4$). (**D**) ELISA was used to measure IgA levels in the supernatants of splenic B cells cultured for 5 days of culture under the same conditions as in (**B**) ($n = 4$). All data are representative of biological replicates at least three independent experiments. Data are represented as the means ± SDs. The significance of differences (**A**) was determined by *t* test, and those (**B**, **C**) were determined using one-way ANOVA with Newman–Keuls post-hoc test. $^{**}P < 0.01$; $^{***}P < 0.005$. Source data are available online for this figure.

© The Author(s)

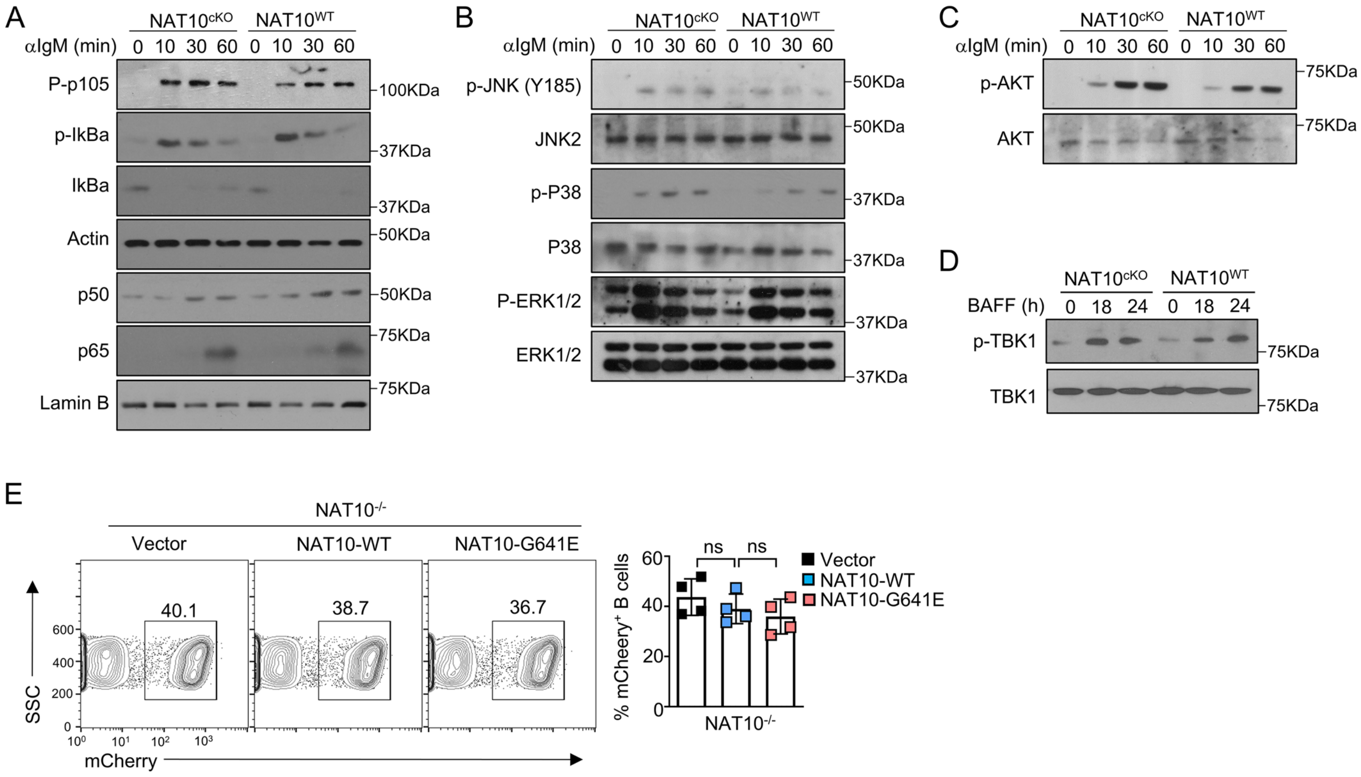

Figure EV5. NAT10 is dispensable for noncanonical NF-κB and MAPKs activation.

(**A–C**) Spleen B cells derived from 6-weeks-old WT and NAT10cKO mice were stimulated as indicated. IB assays detecting the indicated NF-κB, MAPKs and AKT pathways and the loading control Lamin B in the nuclear extracts prepared from the αIgM stimulated WT and NAT10-deficient B cells. (**D**) IB analysis of TBK1 using total cell extracts from WT and NAT10-deficient B cells, stimulated as indicated. (**E**) Immunoassay of $NAT10^{-/-}$ B cells stimulated with LPS (5 μg/ml) for 8 h, followed by infection with a retroviral vector expressing GFP and either WT NAT10 (NAT10-WT) or the NAT10-G641E mutant. Infected cells (GFP^{+}) were analyzed by FACS ($n = 4$). All data are representative of biological replicates at least three independent experiments. Data are represented as the means ± SDs. The significance of differences (**E**) was determined using one-way ANOVA with Newman–Keuls post-hoc test. Source data are available online for this figure.

© The Author(s)
